# The Stress Management and Resiliency Training (SMART) Program Is Associated with Sustained Improvement in Clinician Well-Being: Results from an Observational Cohort Study

**DOI:** 10.3390/ijerph23020161

**Published:** 2026-01-28

**Authors:** Brittany L. Garcia, Maureen A. Craig, Nicole Adams, Elyse R. Park, Michelle L. Dossett

**Affiliations:** 1 Department of Internal Medicine, University of California Davis, Sacramento, CA 95817, USA; blgarcia@health.ucdavis.edu (B.L.G.); macraig@health.ucdavis.edu (M.A.C.); nimadams@health.ucdavis.edu (N.A.); 2 Departments of Psychiatry and Medicine, Massachusetts General Hospital, Boston, MA 02114, USA; epark@mgh.harvard.edu; 3 Center for Healthcare Policy and Research, University of California Davis, Sacramento, CA 95817, USA

**Keywords:** professional burnout, occupational stress, health personnel, meditation

## Abstract

**Highlights:**

**Public health relevance—How does this work relate to a public health issue?**

- Clinician burnout remains a widespread public health challenge linked to reduced clinician well-being, reduced quality and safety of patient care, and increased workforce turnover.
- Evidence-based, multimodal interventions such as the Stress Management and Resiliency Training (SMART) Program offer practical approaches to support clinician well-being.

**Public health significance—Why is this work of significance to public health?**

- The SMART Program is associated with significant and sustained improvements in clinician well-being, perceived stress, burnout, coping, resilience, and self-compassion for at least six months following program completion.

**Public health implications—What are the key implications or messages for practitioners, policy makers and/or researchers in public health?**

- Well-being improvements six months following program completion were most strongly associated with the number of stress-management tools being used by participants.
- Implementation efforts should address structural barriers that limit access for clinicians who may benefit most.

**Abstract:**

Background: Burnout negatively impacts clinicians, patients, and healthcare systems. We examined the immediate and sustained effects of an evidence-based, multi-modal Stress Management and Resiliency Training (SMART) Program on clinician well-being. Methods: Clinicians who registered to participate in the SMART Program were invited to join an observational study and complete questionnaires before the program started, at two months (post-program), and at eight months (six months following program completion). Results: We found significant improvements in well-being, burnout, perceived stress, stress coping, resilience, and self-compassion at 2 months (all *p* < 0.001), with moderate-to-large effect sizes (*d* = 0.57 to 1.0). Significant benefits were maintained at 8 months, with small-to-moderate effect sizes (*d* = 0.41 to 0.65). Exploratory analyses found significant correlations between improvements in well-being from baseline to 8 months and the number of stress-management techniques used at 8 months (*r* = 0.53, *p* < 0.0001) and the number of days on which participants practiced meditation for at least 10 min (*r* = 0.28, *p* = 0.049). Conclusion: Participation in the SMART Program was associated with significant improvements in clinician well-being that persisted six months following program completion and was positively associated with the number of stress-management tools used and meditation practice.

## 1. Introduction

Healthcare professional burnout is associated with reduced quality and safety of patient care, decreased patient satisfaction, decreased clinician job satisfaction and intent to leave, and poorer clinician mental health [1,2,3]. Despite long-standing awareness of this problem, burnout rates in healthcare professionals following the COVID-19 pandemic remain high [4,5]. There is an urgent need to both address institutional/systemic drivers of burnout as well as to better equip clinicians with tools to support them amidst challenging work environments [6].

A number of individual-level support programs have been shown to reduce clinician burnout and stress and improve well-being [7,8,9,10,11]. These programs include skills building in mindfulness, meditation and other mind–body techniques, cognitive behavioral therapy, and/or coping/stress management tools. Most programs teach one or a few techniques, and few programs have studied long-term sustainability of improvements following program completion.

We have previously reported on the adaptation of an evidence-based, multi-modal, mind–body and stress management program, the Stress Management and Resiliency Training Program (SMART Program also known as the SMART-Relaxation Response Resiliency Program [3RP]) [12,13], for healthcare professionals [14,15,16,17]. This program involves training participants to use multiple evidence-based tools aimed at enhancing resiliency to stress (e.g., meditation, mindfulness, and other mind–body tools to decrease the negative physiologic effects of stress; cognitive behavioral therapy to improve stress coping; and adaptive strategies that support positive perspectives, social connections, healthy lifestyle behaviors, empathy, humor, and creativity) [13,14,15]. Prior studies in populations other than healthcare professionals have demonstrated that SMART Program participants experience reduced stress and enhanced stress coping as well as increased mindfulness, optimism, and positive affect [13]. Studies in healthcare professionals have demonstrated significant reductions in clinician stress and improvements in mental and physical health, positive affect, job satisfaction, stress coping, resiliency, and self-compassion [14,16,17]. The objectives of the present study were to examine program effectiveness at another academic medical center, assess measures of well-being and burnout, and investigate the sustainability of improvements in well-being.

## 2. Materials and Methods

### 2.1. Setting

This was a prospective observational study of University of California Davis Health (UCDH) clinicians who voluntarily signed up to participate in the SMART Program. The study was reviewed and granted exempt status by the UC Davis IRB. The SMART Program is an eight session, group-based, multimodal program that teaches participants a variety of different tools (e.g., mind–body, cognitive, positive psychology, and lifestyle) to reduce negative physiologic, cognitive, emotional, and behavioral responses to stress [12]. This program is different from a separately developed program known by the same name [18]. The program was offered as 8 weekly, 1.5 hour (h) synchronous online group sessions which were held 11 times between April 2021 and March 2024.

### 2.2. Participants

The program was advertised to UCDH physicians, advanced practice providers, and nurse leaders via mass emails and direct referrals from the academic and staff assistance program. All clinicians who registered for the program were emailed a link to complete optional surveys hosted on REDCap [19] prior to the beginning of the program (baseline), immediately following program completion (two months), and six months following program completion (approximately eight months after baseline data was obtained). All physicians, advanced practice providers, and nurse leaders who volunteered to enroll in the program were eligible to participate in the study. Study participation was not required to participate in the SMART Program.

### 2.3. Measures

An informational document about the study was included at the beginning of the first set of REDCap questionnaires. If participants selected the consent button and decided to move forward with completing the questions, that constituted consent to participate in the study. At baseline, two months, and eight months, participants were asked to complete the following outcome measures assessed via questionnaires: (1) Expanded Well-Being Index (WBI—score range of −2 to 9, where lower scores represent improved well-being) [20], (2) Perceived Stress Scale-10 (PSS-10—score range of 0 to 40, where higher scores represent greater perceived stress) [21], (3) five items from the Physician Worklife Survey on global job satisfaction (GJS—score range of 5 to 25, where higher scores represent increased job satisfaction) [22], (4) two items from the Measure of Current Status-A assessing perceived stress coping (MOCS-A, items 11 and 12—score range of 0–8, where higher scores represent improved stress coping) [23], (5) two items from the Current Experiences Scale assessing resiliency (CES—items 10 and 18, score range 0 to 8; higher scores represent greater resiliency) [13], and (6) a single question regarding self-compassion (item 8 from the self-compassion scale—score range 1 to 5; lower scores represent greater self-compassion) [24]. Burnout was assessed based on responses to a single question on the WBI. At baseline, participants also completed a short demographic questionnaire assessing their age, sex, race, ethnicity, role, years of practice, and typical number of hours worked per week. Following program completion (at two months) participants were asked two questions about the program’s acceptability by rating on a five-point Likert scale (strongly disagree to strongly agree) the degree to which (1) “The SMART Program is relevant to my life and work” and (2) “The skills taught in the SMART Program are helpful to me”. At eight months, participants were asked about the frequency of their use of SMART Program skills (Supplementary Materials). Program attendance was tracked by the clinician who led the SMART Program groups (MLD).

### 2.4. Statistical Analysis

Descriptive statistics were used to summarize demographic data and participants’ perceptions of the program (SAS v9.4, Cary, NC, USA). Paired samples *t*-tests or McNemar’s test were used to compare changes in questionnaires from baseline to two and eight months. Data were assessed for normality prior to statistical testing. Cohen’s d values were calculated as a measure of effect size. Pearson’s correlation (*r*) was used to assess the relationships between change in WBI scores and number of sessions attended, the number of days in the past month that participants practiced a SMART stress-management technique for any length of time to elicit the relaxation response, the number of days in the past month that participants practiced a technique for at least 10 min continuously to elicit the relaxation response (e.g., meditation, body scan, guided imagery), and the total number of SMART Program tools being practiced. Statistical testing was not corrected for multiple comparisons due to the small sample sizes and pre-specified comparisons based on prior work. Because of how the REDCap survey was set up, there were no measures with partial missing data (either participants completed a measure in its entirety or not at all).

## 3. Results

**Figure 1:**
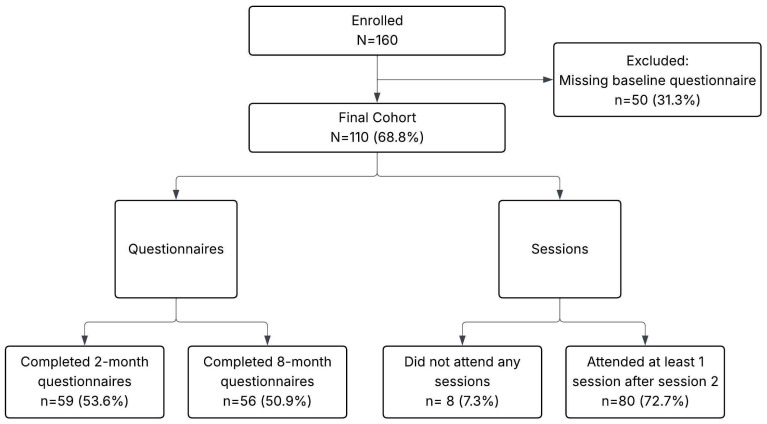
SMART participant flow.

A total of 160 individuals enrolled in the SMART Program (Figure 1). However, due to schedule conflicts and missing baseline data (*n* = 50, 31.3%), our final cohort sample size was *N* = 110. Of those who completed baseline questionnaires, 59 (53.6%) completed at least some of the 2-month follow-up questionnaires and 56 (50.9%) completed at least some of the 8-month follow-up questionnaires. Session attendance was high, with only 8 (7.3%) participants failing to attend any sessions. Sustained attendance was also high, with 80 (72.7%) participants attending at least one session after the second session. The average session attendance for those who attended two or more sessions was 5.3 (*SD* = 2). Forty-seven (42.7%) participants attended at least six sessions.

The overall cohort characteristics of those who completed baseline questionnaires were as follows: participants were, on average, 44.2 (*SD* = 8.7) years old, mostly female (81%), and White (50.9%) or Asian (28%; Table 1 and Supplementary Materials). Participants were largely physicians (69%), with an average of 14.9 (*SD* = 9.7) years of practice, and worked an average of 48.1 (*SD* = 11.6) hours per week.

Paired *t*-tests were used to assess changes in well-being domains from baseline to two- and eight-month follow-up (Table 2). Participants demonstrated significant improvements across nearly all well-being domains at both timepoints. Participants exhibited substantial improvements in well-being (decreased score) at both 2 months (*d* = 0.85, *p* < 0.001) and 8 months (*d* = 0.64, *p* < 0.001). Burnout, assessed using the single-item WBI burnout indicator (with lower scores indicating reduced burnout), also decreased significantly at both timepoints (*d* = 0.63 at 2 months; *d* = 0.47 at 8 months). Perceived stress (for which higher scores indicate greater stress), decreased significantly from baseline to both 2 months (*d* = 1.02, *p* < 0.001) and 8 months (*d* = 0.49, *p* = 0.001), reflecting strong short-term reductions and moderate sustained improvements in stress. Improvements were also observed in domains where higher scores represent better functioning. Stress coping increased significantly at 2 months (*d* = −0.64, *p* < 0.001) and 8 months (*d* = −0.41, *p* = 0.006), indicating better perceived coping effectiveness. Similarly, resiliency improved at both follow-ups (*d* = −0.71 at 2 months; *d* = −0.44 at 8 months), suggesting strengthened resilience over time. Self-compassion also increased significantly at each timepoint (*d* = 0.57 at 2 months; *d* = 0.65 at 8 months). Global job satisfaction (higher scores indicate greater satisfaction) improved significantly at 2 months (*d* = −0.50, *p* < 0.001), but was not significantly different from baseline at 8 months (*p* = 0.21), suggesting initial benefits that were not sustained in the longer term. Collectively, the intervention was associated with broad and significant improvements in well-being, stress, coping, resilience, burnout, and self-compassion at two months, with most benefits maintained at eight months.

Following program completion, participants were asked to rate, on a five-point Likert scale, the degree to which they agreed with two statements. Fifty-five (98%) participants agreed or strongly agreed that the SMART Program was relevant to their life and work. A similar number of respondents (53, 95%) agreed or strongly agreed that the skills taught in the SMART Program were helpful.

**Figure 2:**
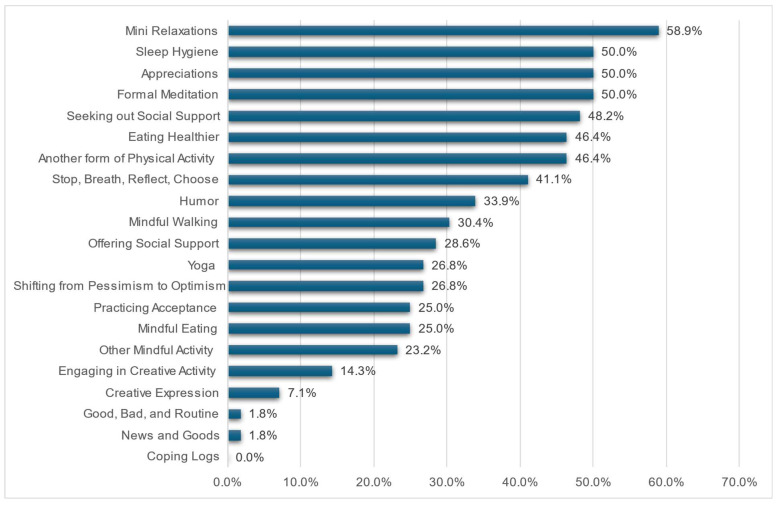
Current practice of SMART Program techniques at 8 months (*n* = 56).

Of those who completed the 8-month follow-up (*n* = 56, 50.9%), the top five stress-management techniques from the program that were still actively practiced included mini relaxations (58.9%), sleep hygiene (50.0%), appreciations (50.0%), formal meditation (50.0%), and seeking out social support (48.2%) (Figure 2). Participants reported practicing techniques to elicit the relaxation response on an average of 12 days (*SD* = 8.4) in the past month and practiced meditation for at least 10 min continuously for an average of 5.7 days over the past month (*SD* = 6.3). Participants were provided with a list of 21 different tools taught in the SMART Program, and on average, were still using 6.4 (*SD* = 3.3) different tools.

Exploratory analyses using Pearson correlation models were used to understand which factors may have contributed the most to changes in WBI scores from baseline to two and eight months (Table 3). Although participants who attended more sessions showed a trend toward improved well-being, this correlation was small and not statistically significant (*r* = 0.21, *p* = 0.13 at 2 months; *r* = 0.24, *p* = 0.09 at 8 months). We also examined the relationship between change in WBI scores from baseline to 8-month follow-up and the number of days in the past month that the participant practiced a technique to elicit the relaxation response (*r* = 0.08, *p* = 0.58) or the number of days participants engaged in ≥10 min of continuous practice of meditation, body scan, or guided imagery (*r* = 0.28, *p* = 0.049). Additionally, we explored the relationship between change in WBI scores from baseline to 8-month follow-up and the number of stress management tools utilized at 8 months and found a moderately strong, statistically significant relationship (*r* = 0.53, *p* < 0.001).

## 4. Discussion

In this prospective observational study of clinicians participating in the SMART Program, we found broad and meaningful improvements across multiple dimensions of well-being, including overall well-being, perceived stress, burnout, coping, resilience, and self-compassion. Improvements were substantial at two months following program completion, with medium-to-large effect sizes across most domains, and were largely maintained at eight months, suggesting that the benefits extended far beyond the intervention period. Notably, there are few studies focused on mind–body programs for healthcare professionals’ well-being that have demonstrated sustained benefits six months following program completion without intervening “booster sessions”.

A meta-analysis of mindfulness-based interventions to reduce stress and burnout in physicians noted moderate effect sizes for stress reduction and small but statistically significant effect sizes for burnout reduction immediately post-program [8]. We found large effect sizes for stress reduction and moderate effect sizes for burnout reduction post-program in this study, possibly related to the greater range of tools provided to clinicians. The effect sizes noted in this study are similar to, or slightly larger than, those noted in prior studies of the SMART Program in healthcare professionals [14,16,17]. Contextual factors such as differences in the setting and the timing of the program may explain the different effect sizes observed. For example, the SMART Program sessions described in this study were delivered virtually for 1.5 h weekly as opposed to 1.5 h every other week in person before the pandemic [14] or 1 h twice a week virtually at the beginning of the pandemic [16]. While a study by Durieux et al. [17] (1.5 h weekly post-pandemic) demonstrated similar significant improvements in resiliency, stress coping, and perceived stress, the current study added six-month follow-up with demonstrated sustained technique utilization that suggests the durability of the intervention’s benefits among clinicians over time. In addition, the current study includes a broader range of specialties and clinicians than our first study [14].

One significant difference in our current findings compared to a prior study relates to changes in burnout. We previously found no significant change in burnout following participation in the SMART Program; however, burnout levels in that cohort were low and the measure used was known to be relatively insensitive to change over a short timeframe [14]. In this study, baseline levels of burnout were higher, and we used a different, single-item binary measure of burnout, which may explain why we saw a significant improvement in burnout that, notably, was sustained over time. We chose to use a single-item measure of burnout to reduce survey burden in participants. Single-item measures of burnout have been validated and found to correlate well with the Maslach Burnout Inventory or other validated measures of burnout [25,26,27]. Despite this difference in observed changes in burnout, the consistency in overall findings across the four studies examining this program for clinicians and conducted in slightly different formats at two different academic medical centers with different interventionists supports the validity of our findings and the utility of this program in supporting clinician well-being.

Although participants improved across nearly all domains, the trajectories varied by measure. The largest short-term gains were observed in perceived stress, global well-being, and resilience, which are key targets of the SMART Program. Improvements in burnout and coping were also observed, though effect sizes diminished somewhat by eight months. Self-compassion continued to improve over time, suggesting that internal self-regulation skills may strengthen with continued practice and integration into daily routines and demonstrating growth enhancement, one of the core components of the SMART Program [12,13]. Only job satisfaction showed an early benefit that was not maintained at eight months, underscoring that organizational or structural factors—rather than individual-level stress-management strategies alone—may be key drivers of long-term occupational satisfaction.

Importantly, participant engagement with the skills taught in the SMART Program was high, with nearly all participants agreeing that the program was relevant and helpful to their work and life. At eight months, participants continued to use an average of six different stress-management techniques, with mini relaxation exercises, sleep hygiene practices, appreciations, formal meditation, and seeking social support being the most frequently reported. These high levels of ongoing use suggest that the program provided practical tools that were identified as meaningful and feasible to incorporate into the daily lives of program participants. This finding is particularly notable given the demanding work schedule of our cohort (approximately 23% worked 60 h or more per week).

Exploratory analyses revealed several insights into the potential mechanisms underlying long-term well-being improvements. Although the number of sessions attended showed small, non-significant associations with well-being improvement at both two and eight months, the quality or intensity of practice appeared more relevant than attendance alone. Specifically, the number of days on which participants engaged in ten minutes or more of continuous elicitation of the relaxation response through meditation or similar practices—reflecting more sustained, intentional engagement—showed a modest but statistically significant correlation with improved well-being at eight months. Furthermore, the strongest association observed in our exploratory analyses was between long-term well-being improvement and the total number of SMART tools participants were using at the eight-month follow-up (*r* = 0.53, *p* < 0.0001). This moderately strong relationship suggests that the breadth of tool use may be a key predictor of long-term benefit. Indeed, the SMART Program is designed to enhance participants’ skills in multiple domains which may interact synergistically to enhance well-being [13,15]. Our findings build upon the results of previous studies focused on using the SMART Program to support healthcare professionals and extend our understanding of how skills training programs may support clinician well-being.

### Strengths and Limitations

This study has several strengths, including longitudinal follow-up up to eight months (six months following program completion) which demonstrated both short- and long-term improvements in overall well-being across several domains. Another strength is its use of multiple validated measures that provide a comprehensive assessment of well-being, burnout, stress, coping, resilience, and self-compassion. High session attendance and continued engagement with SMART tools at eight months further support the program’s acceptability and perceived usefulness.

There are also limitations to our study. As an observational study with no control group, causal inferences cannot be established. Additionally, the sample was predominantly female and from a single academic health system, which may limit the generalizability of the findings. Another substantial limitation is that participation in the SMART Program occurred outside of protected clinical/work time. Thus, individuals who may benefit most from this training may also be the least likely to have the time or flexibility to engage in a voluntary well-being program. As a result, program reach may be skewed toward clinicians already better positioned to participate, limiting generalizability. Indeed, many individuals who wanted to participate had to drop out of the program due to scheduling conflicts. Future studies should evaluate models that integrate protected time or system-level supports to ensure equitable access. Finally, our follow-up response rate (~50%), while comparable to other studies in this population, may inflate our effect size estimates due to missing data and the fact that those who are enthusiastic about the intervention are more likely to complete the program and respond to surveys.

In addition, future studies could incorporate comparison groups and implementation-focused measures to identify potential barriers and facilitators of long-term adoption. Given the sustained practice of the well-being tools at 8-month follow-up, it may be worthwhile to examine outcomes at one year and implement booster sessions to determine whether the benefits can be increased or extended.

## 5. Conclusions

In summary, participation in the SMART Program was associated with significant improvements in well-being, stress, burnout, coping, resilience, and self-compassion, with most benefits sustained at eight months. Exploratory findings suggest that ongoing use of a broad range of stress-management tools may contribute more strongly to lasting improvement than frequency of meditation practice alone. The SMART Program offers a multi-modal, evidence-based toolkit that supports clinician well-being and may be a valuable component of an institution’s well-being offerings.

## Figures and Tables

**Table 1:** SMART participant baseline characteristics (*N* = 110).

Characteristic	Mean (*SD*) or *n* (%)
Age	44.2 (8.7)
Gender	
Male	19 (17.3)
Female	89 (80.9)
Unknown/Prefer not to answer	2 (1.8)
Race	
Asian	31 (28.2)
Black or African American	4 (3.6)
Native Hawaiian/Pacific Islander	1 (0.9)
White	56 (50.9)
Multiracial	4 (3.6)
Other/Prefer not to say/Unknown	14 (12.8)
Hispanic ethnicity	4 (3.6)
Role	
Advanced practice provider	23 (20.9)
Nurse	10 (9.1)
Physician	76 (69.1)
Unknown	1 (0.9)
Years of practice	14.9 (9.7)
Hours worked per week	48.1 (11.6)

**Table 2:** Changes in well-being measures at baseline vs. 2-month follow-up and baseline vs. 8-month follow-up.

	Baseline Mean (SD)	2-Month Follow-Up Mean (SD)	*n*	*p*	Cohen’s d	Baseline Mean (SD)	8-Month Follow-Up Mean (SD)	*n*	*p*	Cohen’s *d*
Well-Being	3.6 (1.9)	2.1 (1.9)	53	<0.001 *	0.85	3.3 (2.2)	1.8 (2.2)	51	<0.001 *	0.64
Burnout	0.88 (0.3)	0.59 (0.5)	56	<0.001 *	0.63	0.84 (0.4)	0.6 (0.5)	55	0.001 *	0.47
Perceived Stress	18.7 (5.3)	14.4 (4.7)	53	<0.001 *	1.02	18.6 (5.6)	15.6 (5.5)	49	0.001 *	0.49
Stress Coping	6.6 (1.6)	7.6 (1.0)	55	<0.001 *	−0.64	6.8 (1.5)	7.6 (1.2)	49	0.006 *	−0.41
Resilience	5.9 (1.4)	7.0 (1.5)	53	<0.001 *	−0.71	6.3 (1.4)	7.0 (1.6)	49	0.004 *	−0.44
Self-Compassion	4.0 (0.8)	3.6 (0.9)	55	<0.001 *	0.57	3.9 (0.8)	3.4 (0.9)j	50	<0.001 *	0.65
Job Satisfaction	17.4 (3.1)	18.6 (3.3)	55	<0.001 *	−0.5	17.6 (3.3)	18.1 (3.9)	51	0.21	−0.18

Figure 2: Not all participants completed each survey; hence, *n* varies for each measure at each timepoint. Score changes were calculated as baseline minus follow-up. Lower scores represent better well-being, reduced burnout, reduced perceived stress, reduced stress coping, reduced resilience, improved self-compassion, and reduced job satisfaction. Paired *t*-tests were used for all domains except for burnout, for which McNemar’s test was used. As Cohen’s *d* is calculated using the follow-up score minus the baseline score, some values are negative. Effect sizes with an absolute value of 0.2–0.49 are considered small, 0.5–0.79 are considered moderate, and 0.8 and above are considered large. * = Statistically significant at α = 0.05.

**Table 3:** Correlations between changes in WBI scores and SMART Program engagement.

Correlation Pair	*r*	*p*	*n*
WBI change (baseline to 2 months) and # of sessions attended	0.21	0.13	53
WBI change (baseline to 8 months) and # of sessions attended	0.24	0.09	51
WBI change (baseline to 8 months) and any length of practice	0.08	0.58	51
WBI change (baseline to 8 months) and ≥10 min of practice	0.28	0.049 *	51
WBI change (baseline to 8 months) and # of stress management tools used	0.53	<0.0001 *	51

* Statistically significant at α = 0.05. # = number.

## Data Availability

The data is available from Michelle L. Dossett upon reasonable request.

## Supplementary Materials

The following supporting information can be downloaded at: https://www.mdpi.com/article/10.3390/ijerph23020161/s1, Table S1: Detailed SMART Participant Baseline Characteristics (N = 110).
