# Supplementary material for: The Stress Management and Resiliency Training (SMART) Program Is Associated with Sustained Improvement in Clinician Well-Being: Results from an Observational Cohort Study
Source: Int J Environ Res Public Health. 2026 Jan 28;23(2):161. doi: 10.3390/ijerph23020161 (PMC12941319; doi:10.3390/ijerph23020161)
Supplement: Supplementary file 1 [file ijerph-23-00161-s001.zip › ijerph-4047452-supplementary.pdf]

Supplementary Materials

Table 1. Detailed SMART Participant Baseline Characteristics (N=110).

Characteristic	Participants (N=110)	
	Mean (SD)	n (%)
**Age**		
25-35 years old	44.2 (8.72)	19 (17.27)
36-46 years old		44 (40.00)
47-57 years old		40 (36.36)
58 years old and above		4 (3.64)
Unknown		3 (2.73)
**Gender**		
Male	-	19 (17.27)
Female		89 (80.91)
Prefer not answer		1 (0.91)
Unknown		1 (0.91)
**Race Ethnicity**		
Multiracial	-	4 (3.64)
Hispanic - Any Race		4 (3.64)
Non-Hispanic Asian		31 (28.18)
Non-Hispanic Black or African American		4 (3.64)
Non-Hispanic Native Hawaiian or Other Pacific Islander		1 (0.91)
Non-Hispanic White		53 (48.18)
Non-Hispanic Other		6 (5.45)
Prefer not to say		5 (4.55)
Unknown		2 (1.82)
**Role**		
Advanced practice provider (i.e., CNS, CNM, CRNA, NP, PA)	-	23 (20.91)
Nurse (RN)		10 (9.09)
Physician (MD, DO)		76 (69.09)
Unknown		1 (0.91)
**Years of Practice**		
5 years or less	14.9 (9.68)	23 (20.91)
6-10 years		19 (17.27)

11-15 years		21 (19.09)
16-20 years		15 (13.64)
21-25 years		14 (12.73)
26 years or more		18 (16.36)
**Hours Worked per Week**		
20-39 hours/week		14 (12.73)
40-59 hours/week	48.1 (11.60)	71 (64.55)
60 or more hours/week		25 (22.73)
